# Evaluation of the Fracture Toughness K_Ic_ for Selected Magnetron Sputtering Coatings by Using the Laugier Model

**DOI:** 10.3390/ma15249061

**Published:** 2022-12-19

**Authors:** Jerzy Smolik, Sylwia Sowa, Joanna Kacprzyńska-Gołacka, Artur Piasek

**Affiliations:** Łukasiewicz Research Network—Institute for Sustainable Technologies, 6/10 Pułaskiego St., 26-600 Radom, Poland

**Keywords:** fracture toughness, nanoindentation method, Berkovich diamond indenter, Laugier model

## Abstract

Nanoindentation is one of the methods that allows for determining the fracture properties of brittle materials. In this article, the authors present the possibility of the fracture toughness coefficient calculation of ceramic-based coatings doped by metal (W, Cr) by using the nanoindentation method with the Berkovich diamond indenter. The mechanical properties of selected coatings, such as hardness and Young’s modulus, were investigated from nanohardness experiments. We analyzed the brittle fracture, which includes changes in hardness (H), Young’s modulus (E), plasticity index H/E and resistance to plastic deformation H^3^/E^2^, enabled the concentration of tungsten and chromium. Due to the size of the indentation and the size of the initial cracks, it is necessary to use Scanning Electron Microscopy (SEM) to observe and measure the indentations made and the generated cracks. For evaluation of the fracture toughness in mode I, the Laugier model was chosen experimentally. The fracture toughness analysis showed that doping with concentrations of 10% W and 10% Cr causes an increase in the fracture toughness for K_Ic_ = 4.98 for TiBW (10%) and K_Ic_ = 6.23 for TiBCr (10%).

## 1. Introduction

The indentation method consists of pressing hard indenters into the test sample and determining their response. These techniques are used more often because they are relatively easy to perform and do not require complicated preparation of the sample and cause only small damage to the surface. First, it was used to determine the hardness, which characterized the resistance of the plastic material to penetration. The study of the mechanical properties led to the development of nanoindentation devices that allow for the application of loads as low as a few mN and provide more information about material behavior than the traditional optical measurement methods [1].

The fracture toughness is one of the most important parameters of technical materials, which often determines their application. In the case of solid materials, the value of fracture toughness is most often described by specifying the critical value of the stress concentration factor, defined as the material constant K_Ic_. This parameter can be interpreted as a critical load, where the initiated process of cracking occurs in the way out of control. The form of the mathematical model used is modified depending on the nature of the cracks. The procedures for determining the experimental values of the K_Ic_ coefficient of solid materials use destructive (impact) methods. It has been described in the applicable standards [2] and consists of the analysis of the fracture process of samples with a properly prepared notch in the three-point bending process [3]. However, there is no clear information on the methodology for testing the fracture toughness of thin coatings produced with Physical Vapor Deposition (PVD) methods. Due to the unavailability of the tested materials in a solid form, which can be used to prepare preparations for tests using impact methods, determining the K_Ic_ value for PVD coatings is very difficult. The mechanical characterization of micro-volume systems such as thin films and coatings [4,5] is a key step in the optimization and development of functional coatings. A lot of attention has been focused on the assessment of the fracture toughness of materials (K_Ic_), including thin coatings, using the nanoindentation method. 

Nanoindentation is a penetration technique that allows for the characterization of the micro-volume of the material by pressing an indenter into the tested material in the direction perpendicular to the surface. Then, the resulting impression is analyzed and the damage of the material accompanying the pressing process, including for example generated cracks, and the analysis of the penetration characteristics, i.e., mutual dependencies of the force acting on the indenter and depth of its penetration. Evans and Charles, in the 1970s, proposed nanoindentation as a method for diagnosing the K_Ic_ coefficient value, which defined the fracture toughness of materials [6]. They described the method of determining the parameter (K_Ic_) for materials, where the initiation and generation of a fracture process is a result of penetration with a Vickers indenter [7]. In the literature, there are known adaptations of various fracture models, including for example the Niihara and Anstis model, for testing the fracture toughness with the penetration method [8,9,10,11,12,13] for various materials ranging from K_Ic_ = 0.74 MPa/m^1/2^–12 MPa/m^1/2^ [14,15,16] with the use of a Vickers indenter. Fewer research results refer to the use of an indenter with Berkovich geometry. The research by Laugier [17], Dukino [18], and Ouchterlony [19] were the first works in this area, which modified the Niihara model by introducing a coefficient, which takes into account the geometry of the indenter used. Despite many studies devoted to the evaluation of the fracture toughness using the nanoindentation method, few of them concern the testing of thin coatings. Using the nanoindentation method results in a limited number of tests for the K_Ic_ parameter for coatings. This limitation results from the great difficulties in interpreting the obtained conclusion, which is a consequence of the appreciable and deliberate heterogeneity of the coatings, including, for example, multilayers and differentiation of phases. This applies to both solid materials and thin coatings, especially coatings used in the environment of cyclically changing external loads. One of the basic technological goals is achieving high fracture toughness for this type of coating, which consequently is the most important criterion for their suitability.

There is no systematization of methodological principle regarding this research, including: The hardness range of the coating, which can use of the method is justified, the possibility of comparing coatings with different parameters (e.g., hardness, thickness), or the influence of the substrate on the obtained effect. The mechanical characteristics of the coatings, such as the fracture toughness, are very important in the process of improvement and better evolution of material solutions for coatings with high tribological effectiveness. Our previous investigation shows that it can be possible to use the Laugier model to assess the fracture toughness of the ceramic coating (TiB_2_) doped with different concentrations of tungsten (W) [20,21].

The aim of the study was to propose a methodology for determining the K_Ic_ coefficient of thin PVD coatings treated using the magnetron sputtering method in the Direct Current (DC) system and examining which load is critical (*P_critical_*) for generating measurable cracks.

This study is focused on nanomechanical characterizations of thin coatings and the determination of their hardness (H), Young modulus (E) and fracture toughness coefficient in mode I (K_Ic_), which can be possible by applying the nanoindentation method. For a better understanding of the methodology, two coatings based on TiB_2_ ceramic with different concentrations of tungsten (TiBW) and chromium (TiBCr) doping were investigated. 

## 2. Materials and Methods

### 2.1. Preparation of Samples

The TiB_2_ coatings with different tungsten and chromium concentrations x = 0, 3, 6 and 10 at.% (Table 1) were prepared on steel substrates using the DC magnetron sputtering method with the original magnetron system designed and manufactured by Łukasiewicz Research Network—Institute for Sustainable Technology in Radom (Ł-ITeE Radom) with Balzer’s pump system. For this purpose, the specially constructed chamber wall was used with localized two circular magnetrons at an angle of 120° to each other. In the two separated deposition processes, we used three targets made on TiB_2_ (99.50% purity) and pure-W (99.95%purity) and pure-Cr (99.95% purity), as reported in the papers [20,21,22]. The dimensions of the targets were as follows: The diameter d = 100 mm and the thickness g = 7 mm. The TiB_2_ dopants with W and Cr coatings were produced in an atmosphere of pure argon (Ar 100%). The series of TiBW coatings were deposited on high-speed steel SW7M and the series TiBCr coatings were deposited on the hot work steel W320. In both cases, the samples have a diameter of 25 mm and a thickness 6 mm. Before the deposition process, the samples were polished by Struers Tegramin-25 (Ł-ITEE, Radom, Poland). The samples were ion-etched in the Ar+ plasma prior to being situated in the process chamber. The substrates were heated up to 300 °C using resistance heaters to obtain better adhesion between the substrate and the coatings. The TiBW coatings were deposited by changes in the source power in the range 25–75 W, but in the case of TiBCr in the range of 70–165 W, respectively. The particular parameters for TiB_2_ coatings with dopants of W and Cr were listed in Table 1. The different source power for the TiB_2_ (2000 W) target in the case of the TiBCr coatings deposition can be explained by the difference in deposition rates of the chromium target compared to the power of the tungsten target. The power of magnetron source was chosen experimentally.

### 2.2. Mechanical Properties of Prepared Samples

The samples from a series of TiBW and TiBCr coatings with different concentrations of dopant elements were examined for hardness testing and Young’s modulus. Evaluating the basic mechanical properties of the tested coatings were carried out using the CSM-TTX/NH2 Nano-Hardness Tester (NHT) by Anton Paar equipped with a Berkovich diamond tip. In agreement with the procedure, the maximum penetration depth was determined by the thickness of 10% of the total thickness of the coatings. To obtain better statistics, 15 measurements of hardness and Young’s modulus were carried out for each of the tested samples. 

### 2.3. The Fracture Toughness by the Laugier Model

For the nanoindentation experiments, the CSM-TTX/NH2 Nano-Hardness Tester (NHT) by Anton Paar, with a diamond Berkovich indenter, was used. The nanoindentation method requires flat, smooth surfaces and indentation equipment. The principle of this method is based on the application of the indenter under a given load and to measure the length of the corresponding cracks generated at the ends of the indentations. The calculation of the fracture toughness is based on the Laugier model, taking into account the two parameters (the load *P* and the length crack (*l*)).

(1) $$K_{\mathrm{Ic}}=X_{v}\cdot {\left(\frac{a}{l}\right)}^{\frac{1}{2}}\cdot {\left(\frac{E}{H}\right)}^{\frac{2}{3}}\cdot \frac{P}{c^{\frac{3}{2}}}$$

where K_Ic_—the fracture toughness coefficient, *X_v_*—indenter geometry factor (for Laugier Equation *X_v_* = 0.016); *E*—Young modulus (GPa); *P*—the applied load (mN); *a*—the distance between the corner and the center of indentation (µm); *l*_1,2,3_—the average crack length, *l* = (*l*_1_ + *l*_2_ + *l*_3_)/3, *c* = *l* + *a*—the sum of *a* and *l*. 

**Figure 1:**
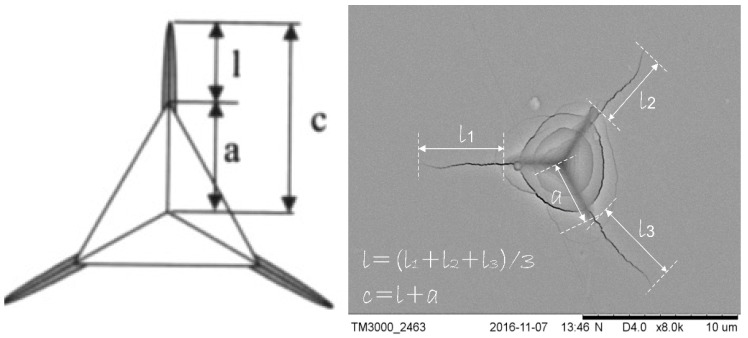
Laugier’s model for determining the fracture toughness K_Ic_ by using the nanoindentation method with the use of a Berkovich indenter for a series of TiBW and TiBCr coatings [20,22].

The analysis of the brittle cracking was carried out in two steps by using a nanohardness tester Anton Paar with a Berkovich diamond indenter. During the testing of TiBW and TiBCr coatings, the critical indenter load was selected, which generated the radial cracks. Indentation tests were performed with different applied loads 50, 100, 200, 300 and 500 mN for each of the tested coatings. The aim of the experiment was also to measure the indenter load, which generated visible cracks for all selected coatings. For each indentation, individual crack length measurements and *l*_n_ were made according to the scheme shown/presented in Figure 1. The selected load is used for making 20 indentations for each coating. For each coating based on 20 indentations, the mean values a and *l* were determined. The value of fracture toughness was calculated according to the Laugier model (Equation (1)). The observation of the crack length measurement in the area of the indentations was carried out by using a scanning electron microscopy SEM Hitachi TM3000 (Ł-ITeE, Radom, Poland). 

## 3. Results

### 3.1. The TiBW and TiBCr Coatings Characterization

In this section, the results of the investigation are presented in detail and compared between two series of coatings with dopants of W and Cr. The concentration and thickness of the brittle fracture cross-section of all coatings obtained by the magnetron sputtering method after deposition were analyzed by scanning electron microscopy. The surface of all tested coatings is characterized by high smoothness and good coherence, which are free of cracks and defects. The thickness of the investigated coatings was in the range of 1.00–1.79 µm. The nanohardness measurements were performed according to the rule of 10% of the coating thickness i.e., max 179 nm. The mechanical properties for the tested TiB_2_ and TiBW and TiBCr coatings are shown in Table 2 and Table 3.

### 3.2. Determination of the Fracture Toughness K_Ic_ for Series of TiBW Coatings

**Figure 2:**
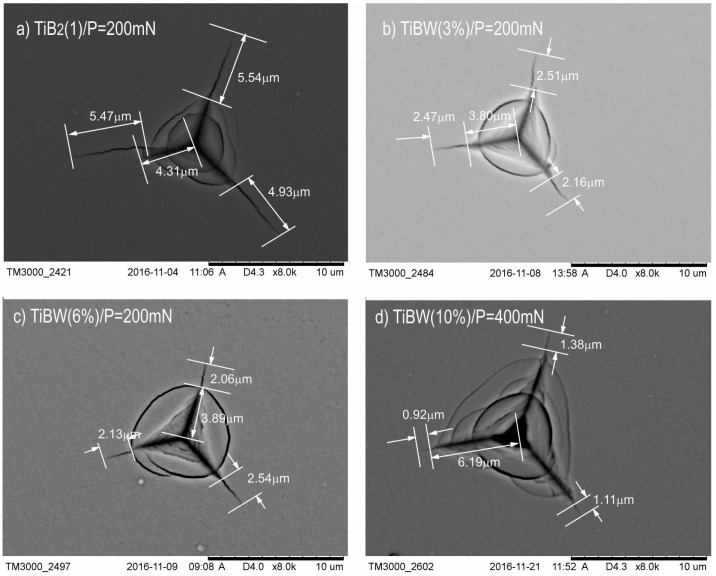
The SEM images of indentations for selected TiB_2_ (1) and TiBW coatings after investigation with different values of applied loading force (**a**) TiB_2_; *P_critical_* = 200 mN; (**b**) TiBW (3%); *P_critical_* = 200 mN, (**c**) TiBW (6%); P = 200 mN and (**d**) TiBW (10%); *P_critical_* = 400 mN.

In the first step, for our investigated coatings, we chose the critical load (*P*_critical_) which generated visible cracks during the selection of the applied loads (from 50 mN–500mN with a step every 50 mN). After the investigation by the Nano-Hardness Tester (NHT), scanning electron microscopy (SEM-Hitachi TM3000) was used to image all the generated cracks. The SEM analysis of indentations for different loads showed, that the indenter load of 200 mN (Figure 2a–c) resulted in the generation of visible and well-measurable cracks from the corner of the indentations for coatings such as TiB_2_, TiBW (3%), TiBW (6%). In the case of TiBW (10%), where the doping of tungsten is the highest, the cracks were visible and well-measurable only with the indenter load of 400 mN (Figure 2d) [20].

According to the adopted methodology, 20 indentations were made for TiB_2_ (1), TiBW (3%), TiBW (6%) coating with the selected indenter load (*P_critical_*), which was 200 mN. Only for the TiBW (10%) coating was the indenter load *P_critical_* = 400 mN. The penetration depth of our TiBW coating was, respectively, h_200mN_ = 1000 nm and h_400mN_ = 1600 nm. The edge lengths for the indentations and the crack lengths *l*_n1_, *l*_n2_ and *l*_n3_ were measured (where n-number of indentations). The average values of *l* and *a* were determined according to Equation *l*_n_ = (*l*_n1_ + *l*_n2_ + *l*_n3_)/3.

(2) $$a=\left(a_{n=1}+a_{n=2}+\ldots +a_{n=20}\right)/20$$

(3) $$l=\left(l_{n=1}+l_{n=2}+\ldots +l_{n=20}\right)/20$$

**Figure 3:**
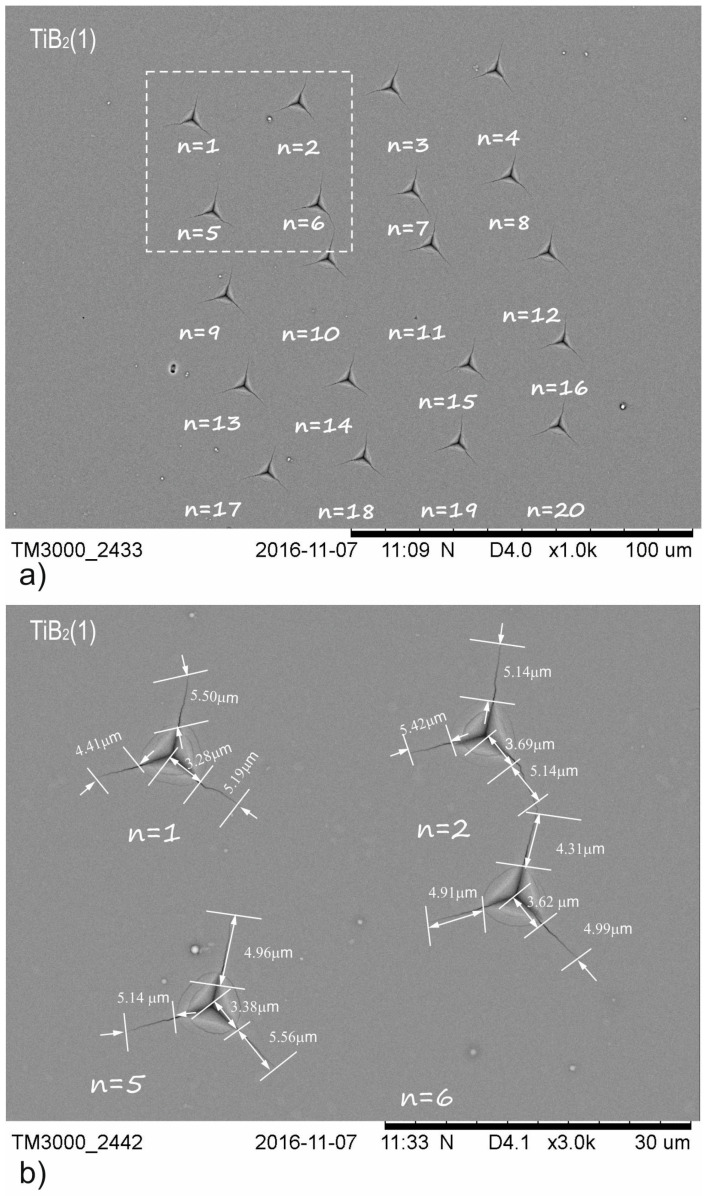
SEM images of groups indentations used for TiB_2_ (1) of fracture toughness analysis: (**a**) series of 20 indentations at a load of *P*_critical_ = 200 mN; (**b**) representation of crack lengths for different indentations where n = 1, 2, 5, 6.

**Figure 4:**
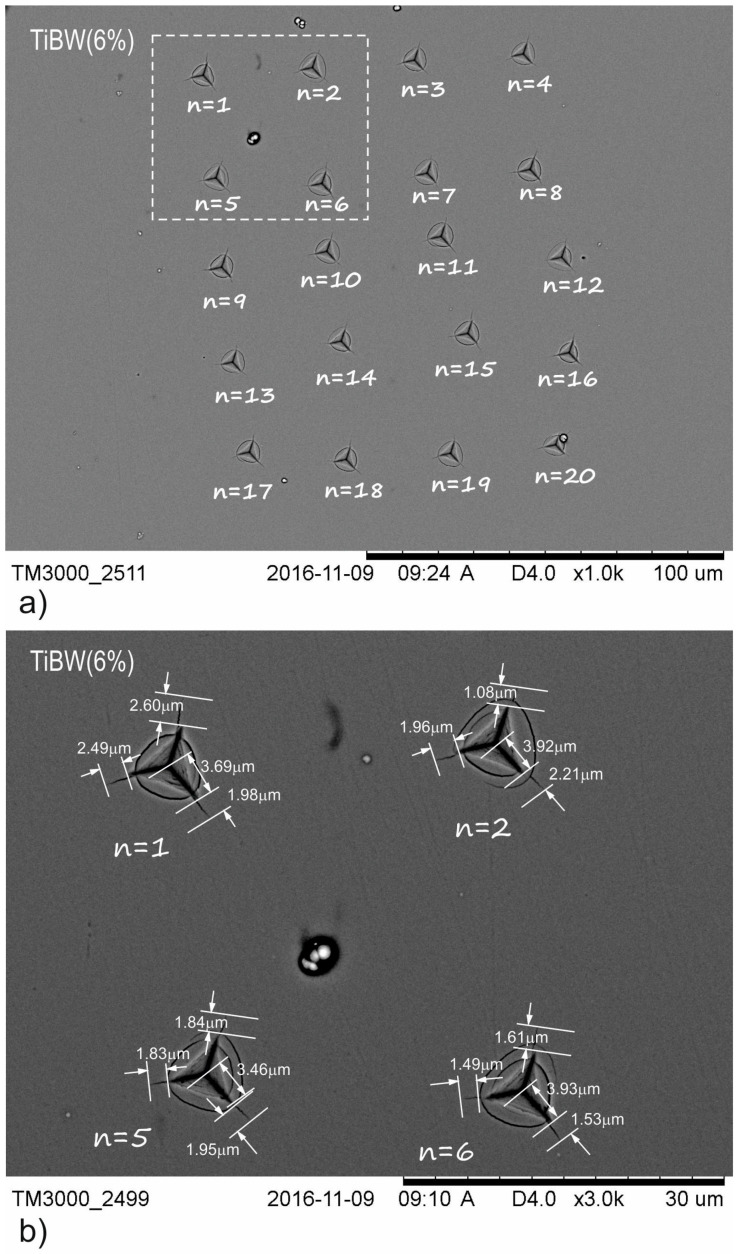
SEM images of groups indentations used for TiBW (6%) of fracture toughness analysis: (**a**) series of 20 indentations at a load of *P*_critical_ = 200 mN; (**b**) representation of crack lengths for different indentations where n = 1, 2, 5, 6 [20].

In accordance with relation to Equations (2) and (3), the average values of *l* and *a* were calculated for the whole series of 20 indentations. The comparison between Figure 3 and Figure 4 shows that increasing the dopants concentration results in a change in crack length (*l*). For TiB_2_ (1), the *l* value is 5.32 µm, for TiBW (3%)the *l* value is 2.12 µm, for TiBW (6%) the *l* value is 2.10 µm and for TiBW (10%) the value is 1.20 µm. The crack lengths for TiBW (10%) are around 7.5 times smaller than for pure TiB_2_ (1). The second observation is that increasing the tungsten concentration influences the length of the *a* value (the distance between the corner to the center of the indentation). The highest average value of *a* was measured for TiBW (10%). Figure 3 and Figure 4 show the example of indentations made for TiB_2_ (1) and TiBW (6%).

### 3.3. Determination of the Fracture Toughness K_Ic_ for Series of TiBCr Coatings

**Figure 5:**
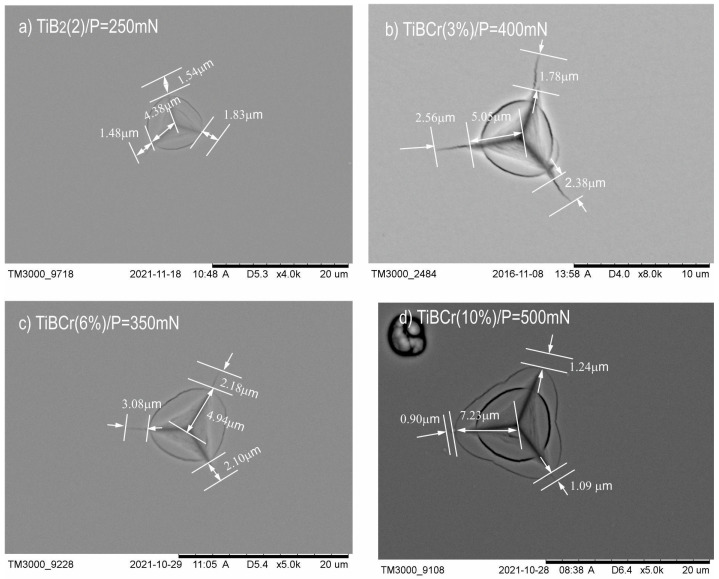
The SEM images of indentations for selected TiB_2_ and TiBCr coatings after fracture toughness experiments with different values of applied loading force (**a**) TiB_2_ (2), *P_critical_* = 250 mN; (**b**) TiBCr (3%), *P_critical_* = 400 mN; (**c**) TiBCr (6%), *P_critical_* = 350 mN and (**d**) TiBCr (10%), *P_critical_* = 500 mN.

The procedure of the measurement for TiBCr coating was similar to the TiBW coating presented in Smolik et al. [20]. First, for the investigation of TiBCr coatings, five different loads were used (50, 100, 200, 300 and 500 mN), but it was not enough. The authors decided to examine samples with more loads, such as 150, 250, 350, and 450 mN to find the critical load. Measuring the lengths of all generated cracks induced by the applied indentation load were analyzed using a scanning electron microscope (SEM—Hitachi TM3000). For the series of TiBCr coatings, the situation was not so easy. For each of the coatings we found different critical loads, where the visible cracks were generated (Figure 5). In the case TiBCr (3%), the critical load, when the cracks were generated was found in the load of *P_critical_* = 400 mN. For TiBCr (6%) coating, the critical load was found at *P_critical_* = 350 mN and for TiBCr (10%) the value of *P_critical_* = 500 mN. For comparison, in the case of the TiB_2_ (2) reference sample, the critical load, when the cracks were generated, was observed in the load of *P_critical_* = 250 mN. The required critical load for generated visible cracks in the case of TiB_2_ (2) is two times smaller than the critical load for TiBCr (10%).

According to the Laugier model, 20 indentations were induced for each coating with critical applied load (P) and then calculated the K_Ic_ values. The value of penetration depth for TiBCr (3%) coatings was respectively h_400mN_ = 1500 nm, for TiBCr (6%) the value of h_350mN_ is 1400 mN and for TiBCr (10%) the value of h_500mN_ is 1700 nm compared with the value of h_250mN_ for TiB_2_ (2) which is 1200 nm. 

**Figure 6:**
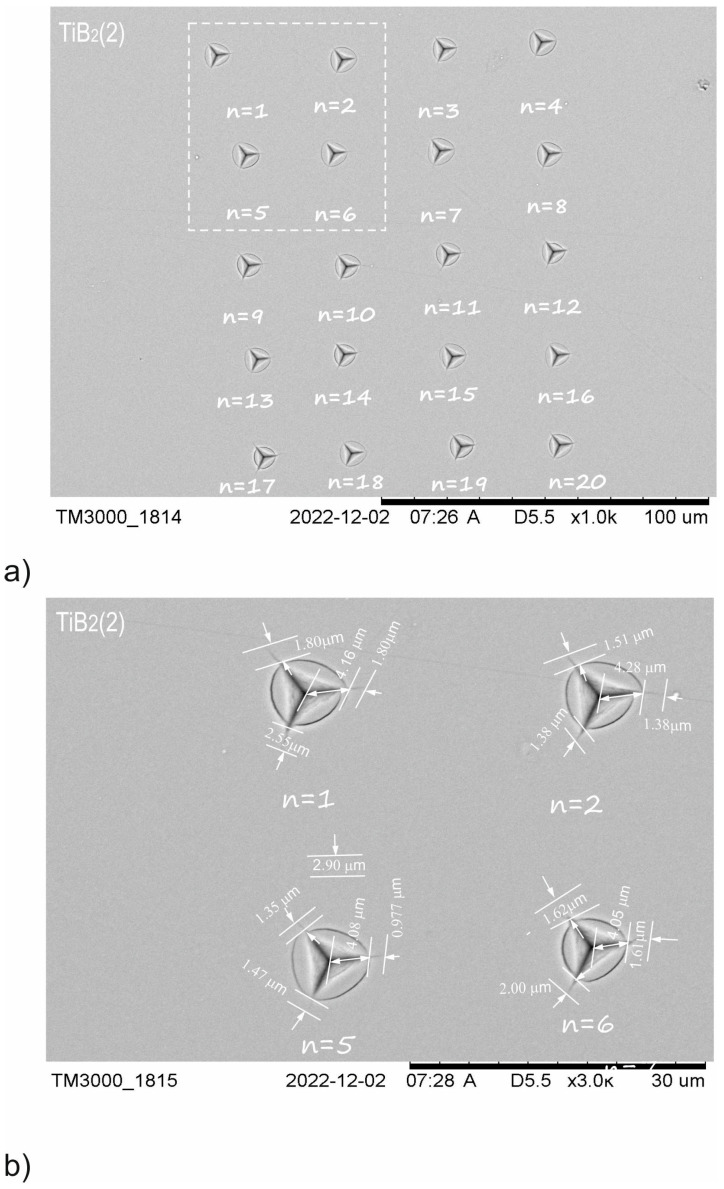
SEM images of groups indentations used for TiB_2_ (2) of fracture toughness analysis: (**a**) series of 20 indentations at a load of *P*_critical_ = 250 mN; (**b**) representation of crack lengths for different indentations where n = 1, 2, 5, 6.

**Figure 7:**
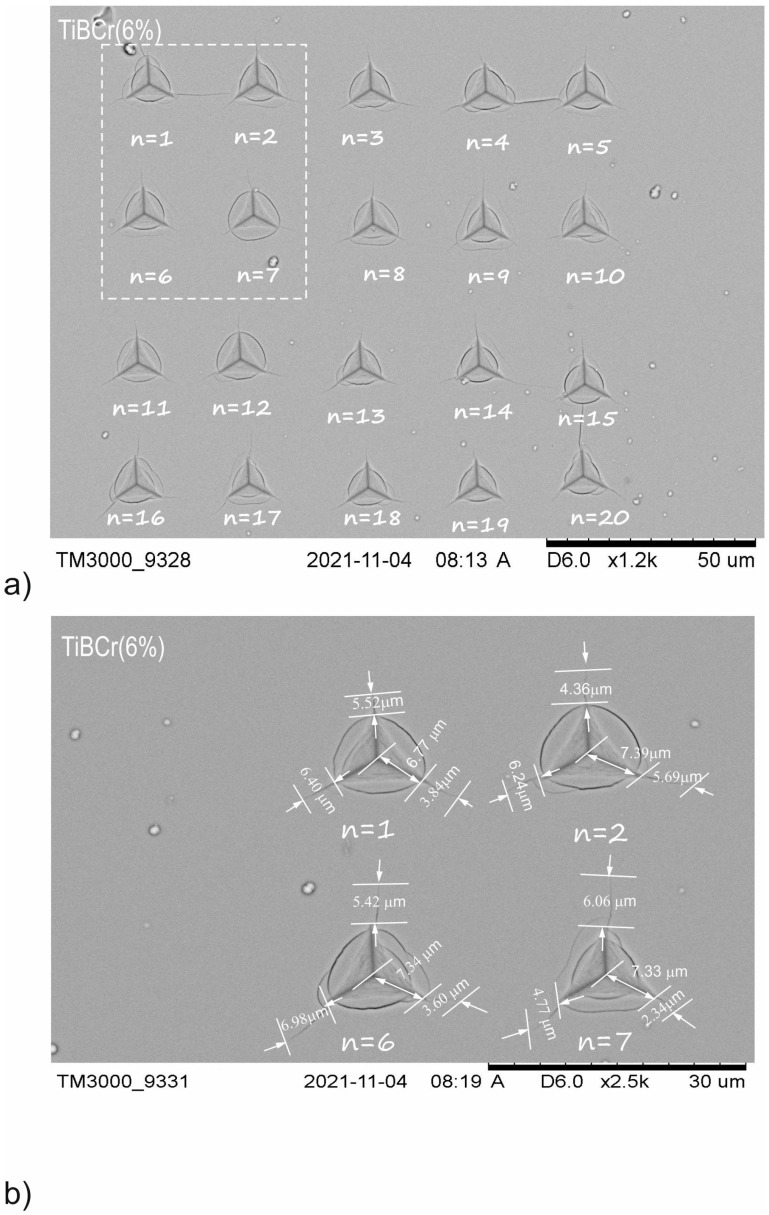
SEM images of groups indentations used for TiBCr (6%) of fracture toughness analysis: (**a**) series of 20 indentations at a load of *P*_critical_ = 350 mN; (**b**) representation of crack lengths for different indentations where n = 1, 2, 6, 7.

The comparison between Figure 3 and Figure 6 shows that increasing dopant concentration results generated a crack length. For example, the average length of generated crack *l* for TiB_2_ (2) is 1.83 µm, for TiBCr (3%)the value of *l* is 1.92 µm, for TiBCr (6%) the value of *l* is 3.04 µm and for TiBCr (10%)the value of *l* is 0.71 µm. The crack lengths for TiBCr (10%) are around 2.5 times smaller than for pure TiB_2_. An additional observation is that the crack length for the coatings from TiBCr series is longer than for coatings from TiBW series. The highest average value of a was calculated for TiBCr (10%). Another important observation is that for TiBW coatings, stable loads are needed for the generation of cracks but in the case of TiBCr coatings, the different critical loads were used for the generation of measurable cracks. The example of indentations made for TiB2 (2) and TiBCr (10%) coatings was presented in Figure 6 and Figure 7.

## 4. Discussion

The presented analysis using the Laugier model showed that the doping of the tungsten (W) or chromium (Cr) in different concentrations affects the change of the value of K_Ic_. It was significantly visible that the fracture toughness increased with increasing concentrations of tungsten and chromium. One of the explanations for this phenomenon is the changing power magnetron during the process. As the authors showed previously in [20], changing the power of tungsten magnetron affects the plasma density and chamber pressure, which can result in nanostructures of coatings. The direction of cracks is changing, and as a consequence, the energy of single cracks can disappear. 

In this article, the authors showed the comparison of TiB_2_ coatings with the doping of chromium and tungsten. Doping the TiB_2_ coating with 3% of chromium presents the increasing plasticity index of coatings H/E and resistance to plastic deformation of coatings H^3^/E^2^ and the fracture toughness coefficient present value of K_Ic_ = 2.55 MPa·m^1/2^ in the critical load *P_critical_* = 400 mN. The fracture toughness for TiBCr (6%) coatings achieves the value K_Ic_ = 1.60 MPa·m^1/2^ in the critical load *P_critical_* = 350 mN. For TiBCr (10%) coatings, the fracture toughness is K_Ic_ = 6.23 MPa·m^1/2^ in the critical load *P_critical_* = 500 mN. This value is around three times higher than for pure TiB_2_ (2) (2.05 MPa·m^1/2^) and 1.25 higher than for TiBW (10%). The very important observation is that, when the chromium and tungsten concentration increase, the critical load for generated visible cracks and the value of Kic coefficient also increase. 

**Figure 8:**
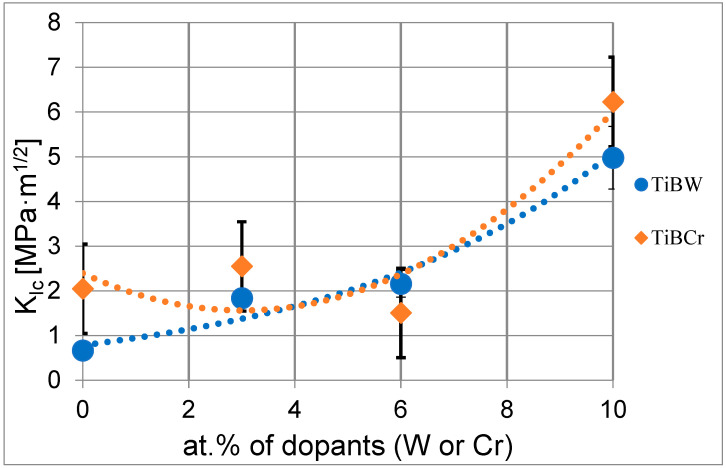
The results of the fracture toughness K_Ic_ analysis for a series of TiBW coatings (blue) and for a series of TiBCr coatings (red) depends on the concentrations of dopants (W or Cr).

Figure 8 shows the plots of changes in the K_Ic_ coefficient for each of the coatings, which shows a characteristic threshold, indicating the value of the load acting on the indenter, when the cracking process is initiated. After exceeding this characteristic threshold, the value of the K_Ic_ coefficient stabilizes. The changes, which are determined for cracks obtained at higher indenter load values depend on the accuracy of the analytical model, the precision of the impressions and the accuracy of microscopic observations and crack length measurements. If the changes in the K_Ic_ coefficient value for the loads acting on the indenter greater than the threshold load responsible for the generation of cracks do not exceed the value of measurement error, it proves a correctly selected measurement methodology. The value of the K_Ic_ coefficient determined for cracks generated at the lowest value of the load acting on the indenter should be considered as the value of fracture toughness.

## 5. Conclusions

The study demonstrated that the brittleness of thin TiB_2_ coatings is increasing with the increasing of dopants tungsten and chromium. The analysis shows that the crack initiation in the group of TiB_2_, TiBCr (3%), TiBCr (6%) and TiBCr (10%) coatings occur when the critical load acting on the indenter (*P_critical_*) is exceeded, which changes for different coatings and different substrate parameters. Based on this, the range of the load acting on the indenter was selected, i.e., *P_critical_* = 200–500 mN. The nanoindentation method, especially the Laugier model, is very convenient for assessing the fracture toughness of coatings based on ceramic. The tungsten and chromium doping increases the value of the K_Ic_ coefficient. 

Our test results showed the complex influence of many factors on the process of indentation and generation of cracks in PVD coatings by pressing a Berkovich indenter. The generation of measurable cracks in the tested coatings is strictly dependent on not only the properties of the coating itself, including the microstructure and phase structure, but also on the properties of the entire substrate-coating system, including its ability to transfer external mechanical loads, which are determined by the property’s elastically—plastic substrates (hardness strength, stiffness) and the thickness of the coating. Based on the conducted analysis, it was shown that the crack initiation in the coating takes place after exceeding the critical load (*P_critical_*), which is also dependent on the parameters of the coating and the parameters of the substrate. This article presents the basis of a methodology for testing the fracture toughness of thin coatings with the use of the penetration method formulated. As a measure of the fracture toughness of the coatings, the K_Ic_ coefficient is interpreted as the critical load, where the initiated fracture process proceeds in an uncontrolled manner as indicated. The correct selection of the analytical model and the simultaneous correct selection of the parameters of the penetration test, including: The geometry of the indenter and the load acting on the indenter, in combination with the information base for various substrate-PVD coating systems, create a very interesting measuring tool enabling quick comparisons to fracture toughness of various substrate-coating systems.

## Figures and Tables

**Table 1:** The deposition parameters by the MS-DC methods for a series of TiBW and TiBCr coatings doped by tungsten and chromium in a concentration of 0, 3, 6 and 10 at % [20,22].

Coatings	Atmosphere	Pressure (Pa)	U_Bias_ (V)	Power of Magnetron TiB_2_/W or Cr
TiB_2_ (1)	Ar 100%	0.5	−50	1000/0
TiBW (3%)			−50	1000/25
TiBW (6%)			−50	1000/50
TiBW (10%)			−50	1000/75
TiB_2_ (2)			−100	2000/0
TiBCr (3%)			−100	2000/70
TiBCr (6%)			−100	2000/100
TiBCr (10%)			−100	2000/165

**Table 2:** The characteristic parameters of thickness, critical load, hardness, Young’s modulus, H/E and H^3^/E^2^ for TiB_2_ (1) and TiBW coatings were used for the calculation of the K_Ic_ coefficient by using the Laugier model [20].

Coatings	Thickness (µm)	Critical Load *P_critical_* (mN)	Hardness H (GPa)	Young’s Modulus E (GPa)	Plasticity Index H/E	Resistance to the Plastic Deformation H^3^/E^2^	*a* (μm)	*l* (μm)	K_Ic_ (MPa·m^1/2^)
TiB_2_ (1)	1.00	200	34.5 ± 2	405 ± 5	0.075	0.239	4.20 ± 0.3	5.32 ± 0.3	0.67 ± 0.1
TiBW (3%)	1.10	200	35.5 ± 2	415 ± 10	0.085	0.259	3.34 ± 0.2	2.12 ± 0.3	1.58 ± 0.2
TiBW (6%)	1.20	200	37.0 ± 2	425 ± 7	0.087	0.280	3.45 ± 0.3	2.10 ± 0.4	1.78 ± 0.3
TiBW (10%)	1.30	400	38.0 ± 3	435 ± 5	0.087	0.289	6.20 ± 0.2	1.20 ± 0.4	4.69 ± 0.7

**Table 3:** The characteristic parameters of thickness, critical load, hardness, Young’s modulus, H/E and H^3^/E^2^ for TiB_2_ (2) and TiBCr coatings were used for the calculation of the K_Ic_ coefficient by using the Laugier model [22].

Coatings	Thickness (µm)	Critical Load *P_critical_* (mN)	Hardness H (GPa)	Young’s Modulus E (GPa)	Plasticity Index H/E	Resistance to the Plastic Deformation H^3^/E^2^	*a* (μm)	*l* (μm)	K_Ic_ (MPa·m^1/2^)
TiB_2_ (2)	1.60	250	34.5 ± 2	410 ± 5	0.084	0.244	4.18 ± 0.3	1.83 ± 0.4	2.05 ± 0.3
TiBCr (3%)	1.73	400	33.3 ± 2	393 ± 9	0.085	0.239	6.13 ± 0.6	1.92 ± 0.6	2.55 ± 0.8
TiBCr (6%)	1.73	350	32.5 ± 1	388 ± 8	0.084	0.228	5.41 ± 0.4	3.04 ± 0.7	1.60 ± 0.4
TiBCr (10%)	1.79	500	30.3 ± 2	383 ± 9	0.079	0.190	7.01 ± 0.3	0.71 ± 0.7	6.23 ± 0.9
